# Investigation of a Salmonellosis Outbreak Caused by Multidrug Resistant *Salmonella* Typhimurium in China

**DOI:** 10.3389/fmicb.2020.00801

**Published:** 2020-04-29

**Authors:** Ying Xiang, Fuxiang Li, Nian Dong, Sai Tian, Haoran Zhang, Xinying Du, Xuan Zhou, Xuebin Xu, Hongxia Yang, Jing Xie, Chaojie Yang, Hongbo Liu, Shaofu Qiu, Hongbin Song, Yansong Sun

**Affiliations:** ^1^Academy of Military Medical Sciences, Beijing, China; ^2^Chinese PLA Center for Disease Control and Prevention, Beijing, China; ^3^Center for Disease Control and Prevention of Southern Theatre Command, Guangzhou, China; ^4^Xingcheng Special Service Recuperation Center of PLA Strategic Support Force, Huludao, China; ^5^Second Medical Center, Chinese PLA General Hospital, Beijing, China; ^6^Shanghai Municipal Center for Disease Control and Prevention, Shanghai, China; ^7^Shanxi Province Center for Disease Control and Prevention, Taiyuan, China

**Keywords:** *Salmonella* Typhimurium, outbreak, multidrug resistant, salmonellosis, phylogenetic analysis, genotypic analysis

## Abstract

The rapid emergence of multidrug resistant *Salmonella* is a global public-health concern as outbreaks in recent years have mostly been caused by multidrug resistant strains. Here, we evaluated an outbreak in China caused by multidrug resistant *Salmonella enterica* serovar Typhimurium (*S*. Typhimurium) by employing an epidemiological and laboratory investigation using conventional methods and whole genome sequencing (WGS). Eleven of the 12 people who participated in a banquet showed gastrointestinal symptoms, and 8*S*. Typhimurium strains were recovered. Isolated outbreak strains showed multidrug resistance (MDR), and decreased susceptibility to ciprofloxacin, a first-line drug recommended by WHO for clinical treatment of intestinal infections. Antimicrobial resistance (AMR) gene analysis indicated that the MDR phenotype of these outbreak strains may be due to the presence of a number of AMR genes, including the *bla*OXA-1 and *bla*TEM-1 β-lactamase genes, which are often plasmid-borne and easily transferred. Further virulence gene analysis indicated that these outbreak strains also carried a large number of virulence genes, including 2 types of *Salmonella* pathogenicity islands (SPI-1 and SPI-2) and many adhesion-related virulence genes. Cluster analysis based on pulse-field gel electrophoresis data and phylogenetic analysis based on WGS revealed that the outbreak clone was closely related to and thus probably derived from local strains. This outbreak caused by multidrug resistant *S.* Typhimurium highlights the need for government improved strategies for the prevention and control of *Salmonella* infections.

## Introduction

*Salmonella* is one of the common pathogens causing sporadic cases or outbreaks of gastroenteritis (Hawker et al., 2019; Jain et al., 2020). According to estimates by the World Health Organization, there were 153 million cases of worldwide non-typhoid *Salmonella* enteric infections in 2010, of which 56,969 were fatal, and about 50% of them were foodborne (Kirk et al., 2015). Data collected by the foodborne disease surveillance system of China from 2006 to 2010, suggested that *Salmonella* was the second most common bacteria causing foodborne outbreaks (Pang et al., 2011). More than 2,600 serotypes of *Salmonella* have been identified (Issenhuth-Jeanjean et al., 2014; Takaya et al., 2019), *Salmonella enterica* serovar Typhimurium (*S*. Typhimurium) is one of the predominant serotypes in many countries (Pieter-Jan et al., 2015; Simpson et al., 2018) and it has held the first or second place in China for many years (Ran et al., 2011; Wang Y. et al., 2017).

In recent years, the antibiotic resistance rate of *S*. Typhimurium has been increasing, making it a global issue of increasing concern that might result in more severe health outcomes (Parisi et al., 2018; Jiu et al., 2020). Some studies have shown that the proportion of multidrug resistant bacteria in *S*. Typhimurium is very high and the most frequently observed antibiotic resistance patterns are the ASSuT (ampicillin, streptomycin, sulfonamides, and tetracycline) and ACSSuT (ampicillin, chloramphenicol, streptomycin, sulfonamides, and tetracycline) tetra- and penta-resistant patterns, respectively (Thung et al., 2016; Mellor et al., 2019; Wang et al., 2019). Multidrug resistant *S*. Typhimurium has brought difficulties to clinical treatment resulting in increased morbidity and mortality.

Outbreaks of *S*. Typhimurium have occurred throughout the world, and most of them were caused by multidrug resistant *S*. Typhimurium (Alt et al., 2015; Folster et al., 2017; Martínez et al., 2017). However, there have been only a few studies on outbreaks caused by multidrug resistant *S*. Typhimurium in China. Existing research has mainly focused on epidemiological investigation and analysis (Liu et al., 2016), rarely analyzing the genetic characteristics of outbreak strains. Here, we report on a multidrug resistant *S*. Typhimurium outbreak in China and examine its molecular characteristics using whole genome sequencing (WGS).

## Materials and Methods

### Outbreak Investigation and Sample Collection

In May 2017, a foodborne salmonellosis outbreak was identified in Changzhi, Shanxi Province. We launched an epidemiological field investigation and concomitant laboratory research. This study was approved by the ethics committee of the Chinese PLA Center for Disease Control and Prevention.

Samples of salmonellosis cases from subjects who attended a rural banquet on May 7, 2017, in Changzhi County were obtained through the Foodborne Disease Surveillance System. The outbreak, which occurred after people attended a rural banquet, was characterized by abdominal pain and diarrhea (5–6 times per day), accompanied by headache, fever, nausea and vomiting. Eleven of the 12 people who participated in the banquet displayed gastrointestinal symptoms and their stool samples were collected. The banquet served a total of 10 dishes, including cooked chicken, cold fried pork liver, pork stir fry with green onions, pork stir fry with garlic, fried Fenpi, ham fry with cucumber, stir fried eggs with tomatoes, fried cucurbita pepo, spicy tofu, bean jelly with cucumber, rice, as well as a bottle of white spirit, nine bottles of beer, and a bottle of soda. Since there were no leftover meals, samples of the remaining ingredients could not be collected. To find the food materials most likely to be contaminated, investigators investigated two markets where food materials used for the banquet were purchased and collected 600 g of cooked chicken, 500 g of bean jelly, and one portion of ham. All samples were placed in sterile plastic sample bags, and kept on ice until transported to the laboratory for testing.

### Isolates and Serotyping

Collected specimens were tested for Salmonella using the following protocol. Stool samples from patients were enriched in Selenite Brilliant Green broth (SBG, CHROMagar, Paris, France) for 16–22 h at 37°C. From each food sample, 25 g were transferred to a sterile plastic bag containing 225 mL of Buffered Peptone Water (BPW, Haibo, Qingdao, China) and incubated at 37°C for 18 h, then each pre-enriched homogenate (1 mL) was aseptically added to 10 mL of lactose broth and incubated at 37°C for 24 h. Consecutively, enriched samples were cultivated on Salmonella-Shigella agar (SS, Haibo, Qingdao, China), xylose-lysine-desoxycholate agar (XLD; CHROMagar, China) and Kligler Iron Agar (KIA, Haibo, Qingdao, China), respectively, and incubated at 37°C for up to 24 h. Suspected bacteria were identified using the commercial biochemical test kit (API 20E system; bioMérieux Vitek, Marcy-L’Etoile, France) according to the manufacturer’s instructions. According to the Kauffmann–White scheme, isolated Salmonella bacteria were serotyped on slides using a microtiter agglutination test for O and H antigens, as described in the manufacturer’s instructions (SSI, Copenhagen, Denmark).

### Antimicrobial Susceptibility Testing

Antimicrobial susceptibility testing was performed by broth microdilution in Sensititre Gram Negative AST Plates for *Salmonella* and *E. coli* (Thermo Fisher Scientific, Inc., West Sussex, United Kingdom) according to the methods of the Clinical Laboratory Standards Institute (2017). The drug-sensitive test plate contained 14 different antibiotic agents: ceftriaxone (CRO), tetracycline (TET), ceftiofur (XNL), cefoxitin (FOX), gentamicin (GEN), ampicillin (AMP), chloramphenicol (CHL), ciprofloxacin (CIP), trimethoprim/sulfamethoxazole (SXT), sulfisoxazole (FIS), nalidixic acid (NAL), streptomycin (STR), azithromycin (AZI), and amoxicillin/clavulanic acid 2:1 ratio (AUG2). An *Escherichia coli* ATCC 25922 strain was used for quality control (Wang J. et al., 2017).

### Pulsed-Field Gel Electrophoresis (PFGE)

Pulsed-field gel electrophoresis (PFGE) of the isolates was carried out following the PulseNet One-day Standardized PFGE Protocol for *Salmonella* with minor modifications (Camarda et al., 2015). Restriction endonuclease digestion was carried out using *Xba*I (Takara, Dalian, China). Electrophoresis was run on a CHEF MAPPER variable angle system (Bio-Rad, CA, United States) with the parameters set at 2.16–63.8 s for 19 h. Images were captured using a Gel Doc 2000 system (Bio-Rad) and converted to TIF format files for further analysis. Captured images were imported into the BioNumerics software (v6.0) database for processing and analysis, and collated with the international standard strain H9218 to calibrate the strip position. Cluster analysis used an unweighted pair group method with arithmetic mean (UPGMA).

### Genome Sequencing and Bioinformatics Analysis

We sequenced eight strains isolated from this outbreak, as well as three other *S*. Typhimurium strains isolated from stool samples of clinical diarrhea patients in Shanxi Province in 2016 and kept in the laboratory, by second generation genome sequencing. Genomic DNA was isolated from overnight cultures using the QIAamp DNA Mini Kit (Qiagen, Hilden, Germany) and paired-end sequences were generated using the Illumina MiSeq platform. Sequence reads were assembled into draft continuous sequences (contigs) using SPAdes software (v3.6.2) (Bankevich et al., 2012).

We downloaded the complete genome sequences of *Salmonella enterica* (602 strains) from the NCBI database in March 2019, including the complete sequence of *S.* Typhimurium (78 strains). Besides, we selected a part of the second-generation sequencing data of S. Typhimurium (32 strains) used in the study of human invasive *S*. Typhimurium pathovariants by Okoro et al. (2012). In order to make the selected strains represent the diversity of S. Typhimurium, we randomly select representative strains in the main branches of their evolutionary tree to locate outbreak strains in this study. Reads were mapped to reference ASM694v2 *S*. Typhimurium for SNP calling using Bowtie with default parameters. SNPs were identified using SamTools (v1.3) (Li et al., 2009). SNPs called in phage regions and repetitive sequences were excluded. Chromosomal SNP alleles were concatenated for each strain to generate a multiple alignment of all SNPs. The resulting alignment was further filtered to remove loci at which alleles were unknown for >95% of isolates (indicating the site is not conserved) and maximum likelihood phylogeny was estimated using RAxML (v8.2.4) (Stamatakis, 2014).

In addition, the 119 strains on the same branch with outbreak strains were analyzed for antimicrobial resistance (AMR) genes and virulence genes. The presence of AMR genes was predicted using the Resistance Gene Identifier (RGI) application of comprehensive antibiotic resistance database (CARD) (Jia et al., 2017), and virulence was determined by BLAST against Virulence Factor Database (VFDB). Heatmap was drawn using ITOL (v4) (Letunic and Bork, 2019).

## Results

### Epidemiological Characteristics of the Outbreak

Investigation of the outbreak incident revealed that there were 12 people who attended the rural banquet on May 7 at 12:30, and 11 of them were infected. The first case presented nausea, vomit and abdominal pain around 10 pm that night, and the last case exhibited symptoms at 17:00 on May 9. Thereby, the shortest, longest and average incubation periods were 10, 53, and 24 h, respectively. May 8 was the peak incidence day (Supplementary Figure S1). Clinical symptoms of all 11 cases included diarrhea (90.9%), abdominal pain (54.5%), nausea (45.5%), fever (81.8%), vomiting (36.4%), and headache (36.4%) (Supplementary Table S1). All patients recovered after receiving symptomatic treatment, and no deaths were recorded. All patients were discharged from hospital before 8:00 on May 15, 2017. Epidemiological investigation of the incident site revealed that both markets held valid Business Licenses, Food Business Licenses and Health Certificates. Only one kitchen knife and one chopping board were employed during the food making process. *S*. Typhimurium was detected in stool samples of 8 among the 11 patients, whereas intestinal pathogens were not detected in the three food samples.

### Antimicrobial Susceptibility

All eight outbreak isolates displayed similar multidrug resistance (MDR) profiles (Table 1). High levels of resistance to TET (minimum inhibitory concentration, MIC; 32 μg/mL), ampicillin (AMP; 32 μg/mL), sulfisoxazole (FIS; 256 μg/mL), nalidixic acid (NAL; 32 μg/mL), and streptomycin (STR; 64 μg/mL), whereas susceptibility to cephalosporin antibiotics were observed. Seven of the 8 strains showed high resistance to trimethoprim/sulfamethoxazole (SXT; 4 μg/mL). Moreover, outbreak strains showed reduced susceptibility to fluoroquinolones and one of them exhibited resistance to CIP.

**TABLE 1:** MIC results of 14 antibiotics for 8 outbreak *S*. Typhimurium strains.

Insolation no.	MICs (ug/mL)^α^													
	CRO	TET	XNL	FOX	GEN	AMP	CHL	CIP	SXT	FIS	NAL	STR	AZI	AUG2
SX17G590	≤0.25	>32	1	4	0.5	>32	16	1	>4	>256	>32	>64	4	16
SX17G592	≤0.25	>32	1	4	0.5	>32	16	4	>4	>256	>32	>64	8	16
SX17G593	≤0.25	>32	1	4	0.5	>32	16	2	>4	>256	>32	>64	4	16
SX17G594	≤0.25	>32	0.5	4	0.5	>32	32	1	>4	>256	>32	>64	8	16
SX17G595	≤0.25	>32	0.5	4	0.5	>32	8	0.5	0.5	>256	>32	>64	4	4
SX17G596	≤0.25	>32	1	4	1	>32	16	1	>4	>256	>32	>64	8	16
SX17G597	≤0.25	>32	2	4	0.5	>32	16	2	>4	>256	>32	>64	8	32
SX17G598	≤0.25	>32	1	2	0.5	>32	8	1	>4	>256	>32	>64	4	>32

*^α^ ceftriaxone (CRO), tetracycline (TET), ceftiofur (XNL), cefoxitin (FOX), gentamicin (GEN), ampicillin (AMP), chloramphenicol (CHL), ciprofloxacin (CIP), trimethoprim/sulfamethoxazole (SXT), sulfisoxazole (FIS), nalidixic acid (NAL), streptomycin (STR), azithromycin (AZI), and amoxicillin/clavulanic acid 2:1 ratio (AUG2).*

### Pulsed-Field Gel Electrophoresis

**FIGURE 1:**
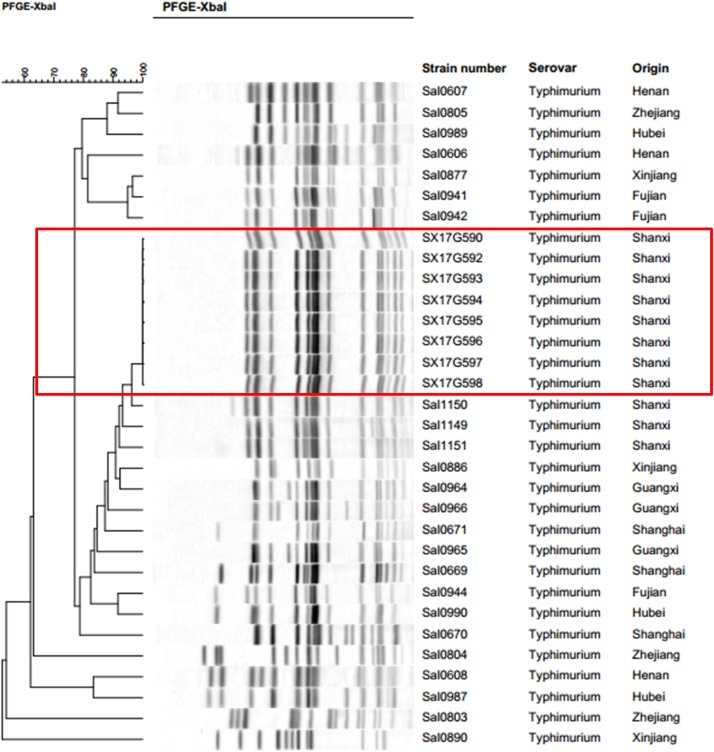
Pulsed-field gel electrophoresis (PFGE) patterns created by digestion with the enzyme *Xba*I. Dendrogram showing the level of genetic relatedness based on the unweighted paired arithmetic averaging method using average linkages and the dice coefficient for *S*. Typhimurium strains.

Pulsed-field gel electrophoresis results of 32 strains of *S*. Typhimurium isolated in eight Provinces, including the eight outbreak strains were clustered together. Results demonstrated that all eight outbreak *S*. Typhimurium strains had identical PFGE bands (Figure 1). The three other strains isolated from the same region as the outbreak strains, were clustered together with the outbreak strains exhibiting a similarity of over 90%.

### Phylogenetic Analyses

**FIGURE 2:**
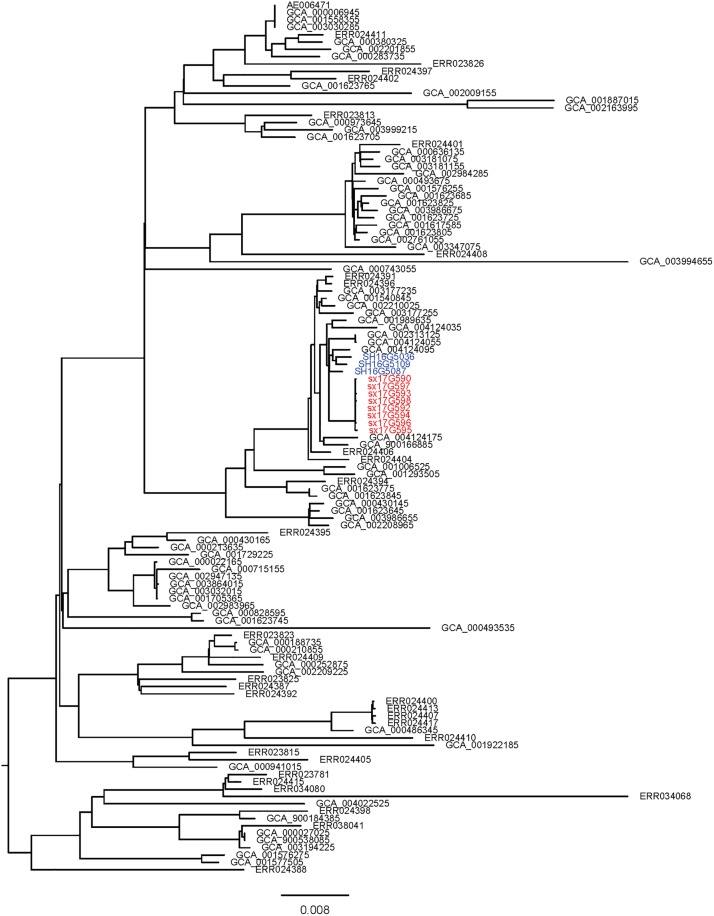
Sub-branch of the outbreak strains. Outbreak strains isolated in this study were marked in red color, and strains kept in our laboratory were marked in blue color.

**FIGURE 3:**
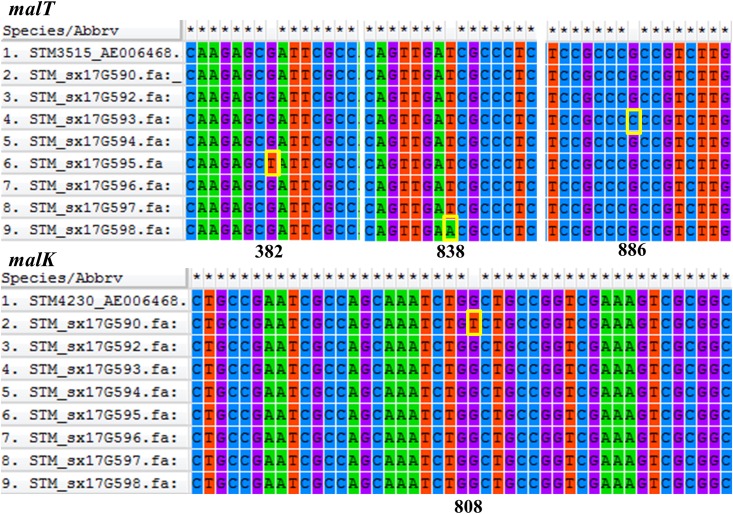
Comparative analysis of the *malT* and *malK* genes. Gene sequences were aligned and visualized using MEGA6 software. Position of each gene sequence of outbreak strains was compared to the ASM694v2 reference genome of *S*. Typhimurium shown in the first row of each alignment. The mutation sites of the outbreak strains were marked with yellow boxes.

Phylogenetic analyses of 645 strains showed that 11 of the self-test strains were all located in the blue branch (Supplementary Figure S2). Therefore, we performed a second phylogenetic analysis of 119 strains located on blue branches, all of which were *S.* Typhimurium and generated a phylogenetic tree with 11487 SNP loci (Figure 2). The eight outbreak strains were closely clustered together with their core genome differing only by a few SNPs (*n* = 21) (Table 2), suggesting that these strains originated from a single clone. Difference in the 21 SNP loci resulted in 9 synonymous mutations and 12 non-synonymous mutations. These non-synonymous mutations were concentrated on four genes, one of which was a putative response regulator gene, one was a putative cytoplasmic protein gene, and the other two were mal family genes, the *malK* and *malT* genes, respectively. MalK, maltose/maltodextrin ABC transporter ATP-binding protein, can control negatively the activity of MalT, a HTH-type transcriptional activator dedicated to the maltose regulon (Davidson and Alvarez, 2010). One outbreak strain harbored a point mutation in the *malK* gene, and three outbreak strains harbored point mutations in three different sites of *malT* gene, respectively (Figure 3). Mutations in these two genes affect the strain’s metabolism to maltose.

**TABLE 2:** The SNP differences detected among the isolates from this outbreak.

SNP position	SNP	Substitution^a^	Isolates	Product
391265	T → G	Val70Gly	SX17G596	Putative response regulator
2339742	A → G	Tyr59His	SX17G595	Putative cytoplasmic protein
2339755	G → A	Synonymous	SX17G595	Putative cytoplasmic protein
2339785	G → C	Synonymous	SX17G595	Putative cytoplasmic protein
2339787	T → C	Thr74Ala	SX17G595	Putative cytoplasmic protein
2339922	T → G	Ile 119Leu	SX17G598	Putative cytoplasmic protein
2339933	A → G	Leu123Pro	SX17G598	Putative cytoplasmic protein
2340010	A → T	Synonymous	SX17G590, SX17G598	Putative cytoplasmic protein
2340022	T → C	Synonymous	SX17G590, SX17G598	Putative cytoplasmic protein
2340025	G → A	Synonymous	SX17G590, SX17G598	Putative cytoplasmic protein
2340028	T → G	Asp155Glu	SX17G590, SX17G598	Putative cytoplasmic protein
2340039	T → C	Asp158Asn	SX17G590, SX17G598	Putative cytoplasmic protein
2340073	A → C	Synonymous	SX17G590, SX17G598	Putative cytoplasmic protein
2340076	A → G	Synonymous	SX17G590, SX17G598	Putative cytoplasmic protein
2340139	G → A	Synonymous	SX17G590	Putative cytoplasmic protein
2340159	T → C	Glu198Lys	SX17G590, SX17G598	Putative cytoplasmic protein
2763434	G → T	Synonymous	SX17G590, SX17G597	Gifsy-1 prophage protein
3677128	G → T	Arg128Leu	SX17G595	Transcriptional activator of the mal genes
3677584	T → A	Ile280Asn	SX17G598	Transcriptional activator of the mal genes
3677632	G → C	Arg296Pro	SX17G592	Transcriptional activator of the mal genes
4451690	G → T	Cys270Trp	SX17G590	Maltose transport protein

*^*a*^The SNP was detected by mapping the reference genome *S*. Typhimurium ASM694v2.*

### Genotypic Characterization of Antimicrobial Resistance

**FIGURE 4:**
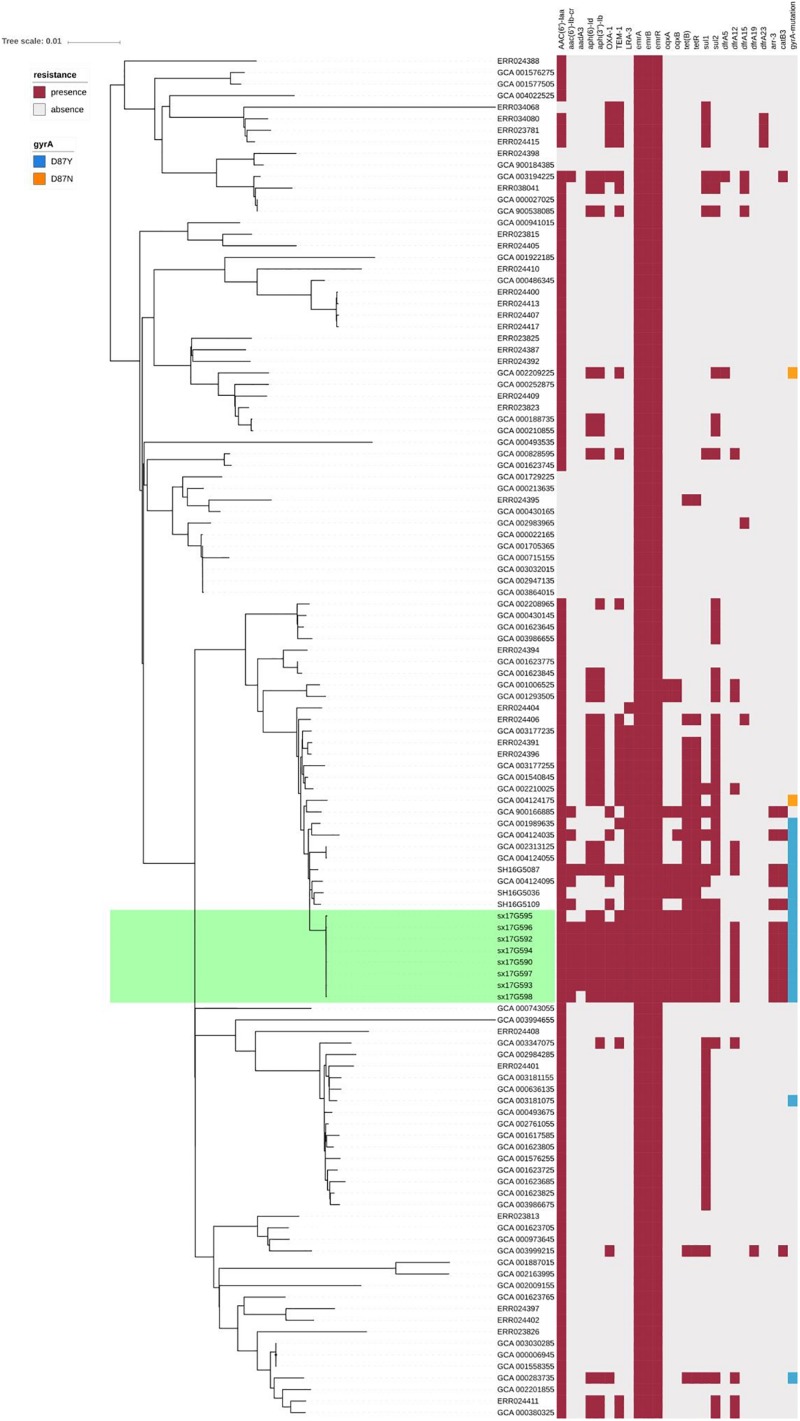
AMR gene groups detected in each genome sequence at more than 50% coverage and 75% identity using BLAST (BLASTn). Presence and absence of AMR genes were represented by dark red and light gray colors, respectively. Presence of the *gyrA* (Asn87Asx) and *gyrA* (Asn87Tyr) point mutations were represented by dark blue and dark orange colors, respectively.

We performed AMR genes analysis on 119 strains in Figure 2, which were extracted from whole genome sequencing (WGS) analysis. Results presented in Figure 4 revealed that all outbreak strains harbor similar AMR genes. All eight outbreak stains carried 14 AMR genes, including two groups of fluorquinolone resistance genes, namely *oqxA* and *oqxB*, two groups of TET resistance genes, namely *tet(B)* and *tetR*, two groups of sulfonamide resistance genes, namely *sul1* and *sul2*, three groups of aminoglycoside resistance genes, namely *AAC(6’)-Iaa*, *APH(3”)-Ib* and *APG(6’)-Id*, three groups of erythromycin resistance genes, namely *ermA*, *ermB* and *ermR*, and two groups of β-lactamase genes, namely *bla*TEM-1 and *LRA3*, as well as contained the *gyrA* (Asn87Asx) point mutation. In addition, seven out of the eight outbreak strains were found to carry the *AAC(6’)-Ib-cr* fluoroquinolone resistance gene, *catB3* phenicol resistance gene, *arr-3* rifampicin resistance gene, *bla*OXA-1 β-lactam resistance gene and *dfrA12* trimethoprim resistance gene. Lastly, six outbreak strains carried the *aadA3* aminoglycoside resistance gene.

### Virulence Genes Analysis

**FIGURE 5:**
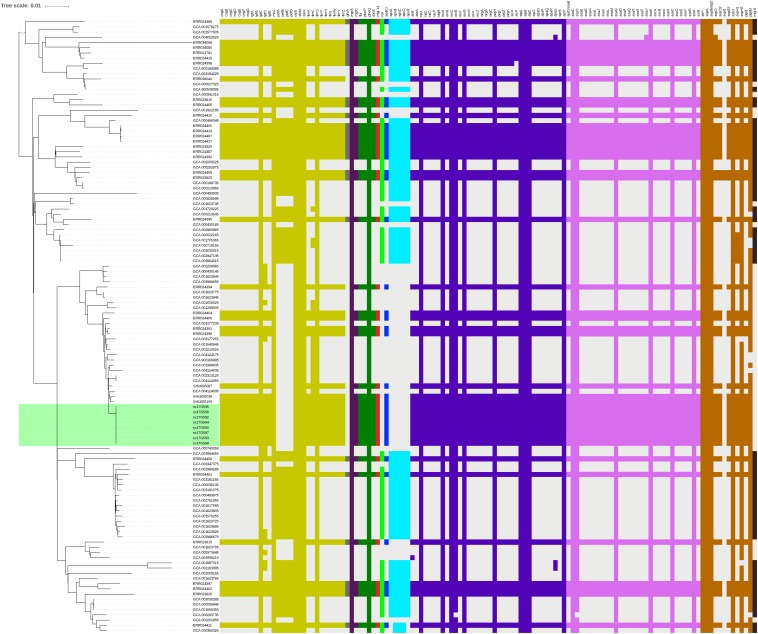
Virulence gene analysis of *S*. Typhimurium strains. Heatmaps were generated by aligning the draft genome sequence of each stain to the virulence gene database. Presence of virulence genes is represented by different color boxes with different kinds of virulence genes, whereas absence of virulence genes is represented by a light gray box. The following gene groups were represented by the respective color-coded boxes, as follows: adherence-related genes by dark yellow, antivirulence-related genes by dark gray, magnesium uptake-related genes by dark purple, regulation-related genes by dark green, resistance to antimicrobial peptides genes by dark red, serum resistance genes by light green, stress protein genes by dark blue, toxin related-genes by light blue, SPI-1 genes by blue-violet, SPI-2 genes by plum, macrophage-inducible genes by black and other virulence genes by brown.

Virulence gene analysis on 119 strains in Figure 2, we found that these virulence genes were exactly the same in all outbreak strains (Figure 5). The identified virulence genes included the *csgA-G* curli fiber specific gene, *lpfA-E* long polar fimbriae gene, *fimA-Z* type 1 fimbriae related gene, as well as the misL, *ratB*, *shdA* and *sinH* genes, which encode various virulence factors involved in adherence. All outbreak strains had two types of *Salmonella* pathogenicity islands (SPI-1 and SPI-2), and four types of regulatory function-related genes (*fur*, *phoP*, *phoQ*, *rpoS*), but lacked toxin-related genes (*cdtB*, *spvR*, *spvA-D*) and the *pefA-D* plasmid-encoded fimbriae gene.

## Discussion

Here, we report on an outbreak case of *S*. Typhimurium infection. Epidemiological surveys showed that all 11 people exhibiting gastrointestinal symptoms attended the same banquet and presented with symptoms within 3 days of attending the banquet. All isolated outbreak strains showed similar antibiotic resistance patterns. PFGE is the most commonly used molecular subtyping method for outbreak detections and surveillance because it has been used as the method of choice for bacterial “fingerprinting.” PFGE results demonstrated that all eight strains bore completely identical bands, which is considered the gold standard condition when determining an outbreak. However, studies have now confirmed that WGS analysis provides better resolution than PFGE in outbreak determination (Deng et al., 2016; Oakeson et al., 2018). In the phylogenetic analysis, all eight strains were tightly clustered together, with only 21 single nucleotide polymorphism (SNP) loci found to be inconsistent. These results confirmed that this incident was indeed an outbreak of *S*. Typhimurium infection.

*Salmonella* Typhimurium enteritis can occur all year round and is generally acute, with an incubation period of 8 to 24 h. Incidence rates, however, are higher in summer and autumn. In this study, the investigated outbreak occurred close to the summer season, and incubation period differed significantly among the 11 patient, ranging from 10 to 53 h. Most treatments applied were symptomatic-based, and due to the timely treatment all patients recovered within 9 days. Since there was no food left, the epidemiological investigation could not identify the source of contamination on the food served, but discovered that only one kitchen knife and one chopping board for both raw food and cooked food were found in the kitchen, which is a common phenomenon in rural areas of China. We speculated that this practice was the main cause of this outbreak. On the bright side, this outbreak made the local government realize that it should increase publicity and education on food-borne diseases in rural areas and improve the health awareness of residents. Although we did not trace the source of the outbreak stains, we discovered a close relationship between the outbreak strains and local strains isolated from Shanxi Province. PFGE cluster analysis and WGS phylogenetic analysis showed that the eight outbreak strains were most closely related to the three other strains isolated from Shanxi Province. Moreover, these three strains were isolated on 2016, 1 year ahead of the outbreak incident. Therefore, we speculated that this outbreak was probably caused by a local strain.

All eight isolated strains showed an ASSuT tetra-resistant pattern consistent with the results of resistance genes analysis. The *bla*TEM-1, *LRA3* and *bla*OXA-1 genes carried by the outbreak strains conferred resistance to AMP, the *AAC(6’)-Iaa*, *APH(3”)-Ib*, *APG(6’)-Id* and *aad3* genes conferred resistance to STR, the *sul1* and *sul2* genes conferred resistance to FIS, and the *tet(B)* and *tetR* genes conferred resistance to TET. In addition, outbreak strains also showed resistance to NAL. These five antibiotics were the most common antibiotics for which *S*. Typhimurium exhibited resistance, especially in China (Wang J. et al., 2017; Liang et al., 2018; Khan et al., 2019; Mellor et al., 2019). NAL belongs to the first generation of quinolone antibiotics, and many intestinal infecting bacteria have developed resistance to it. Currently, this antibiotic is rarely used in clinical practice. On the other hand, as a third generation quinolone antibiotic, CIP has broad-spectrum antibacterial activity and is the first-line drug for clinical treatment of intestinal infections. At present, the proportion of intestinal bacteria resistant to CIP is low (Moreira da Silva et al., 2017; Barour et al., 2019). Resistance mechanisms to quinolone antibiotics include changes in target sites, the role of active efflux systems, changes in membrane permeability, as well as plasmid-mediated acquired resistance (Fàbrega et al., 2014; Aldrich et al., 2019). Changes in target sites alone can lead to antibiotic resistance. In this study, all outbreak strains contained the gyrA(Asp87Tyr) point mutation and carried the *oqxA*, *oqxB* and *AAC(6’)-Ib-cr* genes. These were the reasons for their acquired resistance to NAL and decreased sensitivity to CIP. Moreover, one of the strains even showed resistance to CIP, a finding that should be taken seriously (Correia et al., 2017). Outbreak strains also carried the *bla*OXA-1 and *bla*TEM-1 β-lactamase genes, which are often plasmid-borne and easily transferred, thus causing horizontal spread of antibiotic resistance (Li et al., 2007; Rodríguez et al., 2008).

*Salmonella* has many virulence factors that play an important role in overcoming the immune defense mechanisms of the host during infection. The vast majority of important gene-encoded virulence factors are mainly located on pathogenicity islands (SPIs), which are highly conserved in *Salmonella* (Marcus et al., 2000; Michael et al., 2006). All eight outbreak strains carried both SPI-I and SPI-II. SPI-I and SPI-II encode 2 type-III secretion systems required to translocate at least 28 effector proteins from vacuolar-resident bacteria into host cells (Jennings et al., 2017). The outbreak strains carried a series of genes associated with adhesion (*lefA-E*), colonization (*RatB* and *SinH*) and biofilm formation (*csgA-G*), which promote invasion and survival of strains in unsuitable environments (Desai et al., 2019; Elkenany et al., 2019). We speculated that this was one of the main reasons enabling these strains to generate outbreaks in the population.

## Conclusion

We described an outbreak in China caused by *S*. Typhimurium strains carrying both multidrug-resistance genes and multiple virulence factors. The emergence and spread of these outbreak strains might necessitate government improved strategies for the prevention and control of *Salmonella* infections. Moreover, it is important to continuously monitor the prevalence of *Salmonella* strains and any changes in antibiotic resistance patterns and virulence, both nationally and internationally.

## Data Availability Statement

The complete nucleotide sequences of strains SX17G590, SX17G592, SX17G593, SX17G594, SX17G595, SX17G596, SX17G597, and SX17G598 were submitted to GenBank of NCBI under the accession number PRJNA578823.

## Ethics Statement

The studies involving human participants were reviewed and approved by the Ethics Committee of the Chinese PLA Center for Disease Control and Prevention. Written informed consent from the participants’ legal guardian/next of kin was not required to participate in this study in accordance with the national legislation and the institutional requirements.

## Author contributions

YX wrote the main manuscript and fully participated in all experiments. SQ designed the study. ND, XD, and XZ participated in data collection. XX and HY participated in the specimen collection. ST, HZ, and JX participated in all experiments. FL, CY, and HL contributed to the bioinformatics data analysis. HS and YS gave final approval of the version to be submitted. All authors made substantial contributions to preparation and submission of manuscript.

## Conflict of Interest

The authors declare that the research was conducted in the absence of any commercial or financial relationships that could be construed as a potential conflict of interest.

## Supplementary Material

FIGURE S1 Epidemic curve of the *S*. Typhimurium outbreak by day of symptom onset.

FIGURE S2 Phylogenetic tree of *S*. Typhimurium. The branch where the outbreak strains are located was marked in blue color, and 11 of the self-test strains were marked in red color. Reads were mapped to the ASM694v2 reference genome of *S*. Typhimurium.

TABLE S1 Epidemiological characteristics of 11 incident cases in the outbreak case.
