# Supplementary figures and images for: Investigation of a Salmonellosis Outbreak Caused by Multidrug Resistant Salmonella Typhimurium in China
Source: Front Microbiol. 2020 Apr 29;11:801. doi: 10.3389/fmicb.2020.00801 (PMC7200987; doi:10.3389/fmicb.2020.00801)

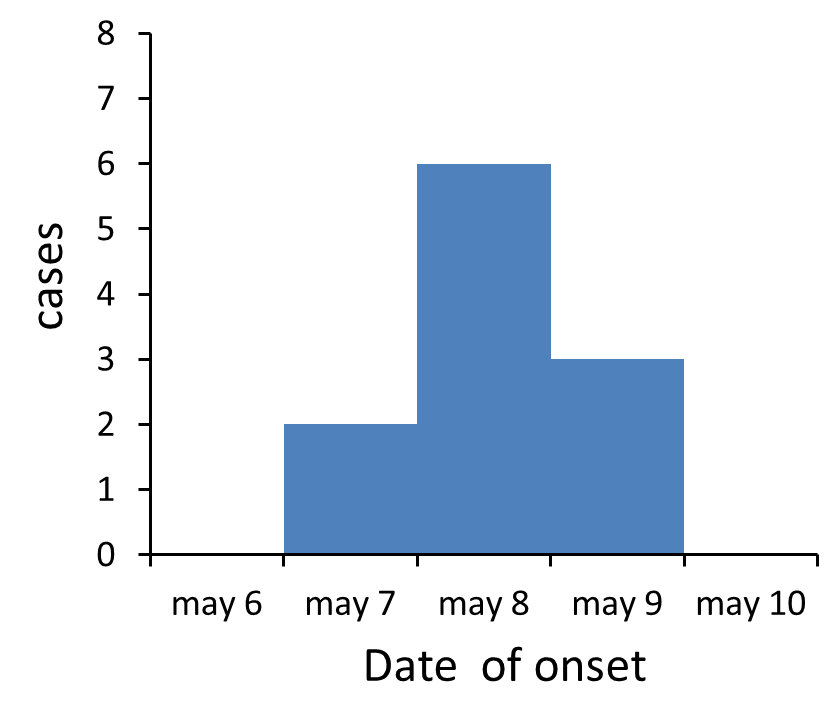

Supplement: FIGURE S1 — Epidemic curve of the S. Typhimurium outbreak by day of symptom onset. [file Image_1.TIF]

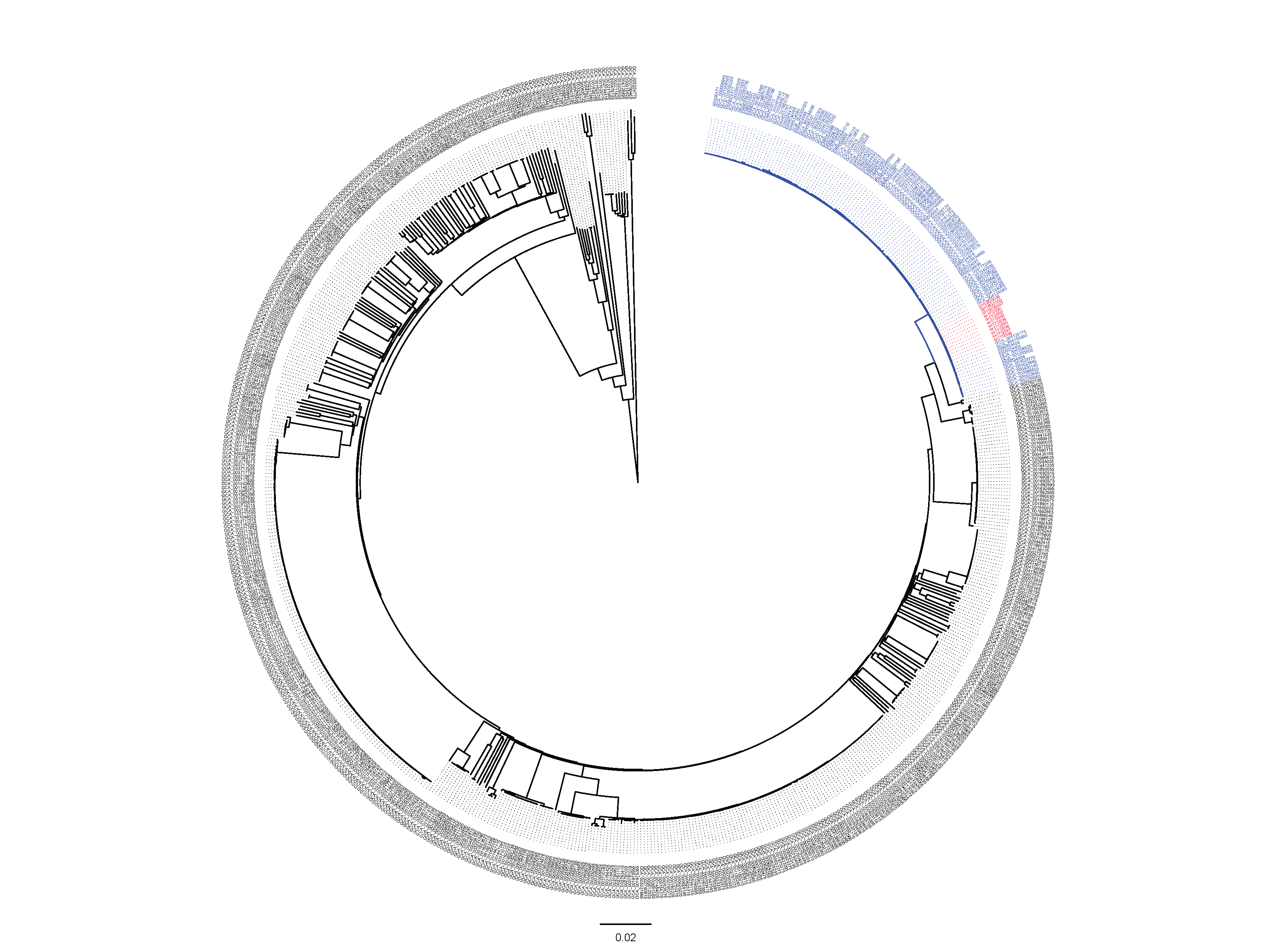

Supplement: FIGURE S2 — Phylogenetic tree of S. Typhimurium. The branch where the outbreak strains are located was marked in blue color, and 11 of the self-test strains were marked in red color. Reads were mapped to the ASM694v2 reference genome of S. Typhimurium. [file Image_2.TIFF]
