# Supplementary material for: Investigation of a Salmonellosis Outbreak Caused by Multidrug Resistant Salmonella Typhimurium in China
Source: Front Microbiol. 2020 Apr 29;11:801. doi: 10.3389/fmicb.2020.00801 (PMC7200987; doi:10.3389/fmicb.2020.00801)
Supplement: TABLE S1 — Epidemiological characteristics of 11 incident cases in the outbreak case. [file Table_1.DOCX]

**Supplementary Table S1│**Epidemiological Characteristics of 11 Incident Cases in the Outbreak Case

| Characteristics of cases | patients | Constituent ratio (%) |
| --- | --- | --- |
| Gender |  |  |
| Male | 4 | 36.4 |
| Female | 7 | 63.6 |
| Age group(year) |  |  |
| 0~10 | 3 | 27.3 |
| 11~20 | 1 | 9.1 |
| 21~30 | 0 | 0.0 |
| 31~40 | 2 | 18.2 |
| 40~50 | 2 | 18.2 |
| >50 | 3 | 27.3 |
| Symptoms/signs |  |  |
| Diarrhea | 10 | 90.9 |
| Abdominal pain | 6 | 54.5 |
| Nausea | 5 | 45.5 |
| Fever | 9 | 81.8 |
| Vomiting | 4 | 36.4 |
| Headache | 4 | 36.4 |
